# Case Report of Presumed (In)voluntary Capsaicin Intoxication Mimicking an Acute Abdomen

**DOI:** 10.1155/2020/3610401

**Published:** 2020-06-23

**Authors:** Simona Koprdova, Christine Schürmann, Dirk Peetz, Thomas Dürbye, Frank Kolligs, Herbert Koop

**Affiliations:** ^1^Department of General Internal Medicine and Gastroenterology, Helios Klinikum Berlin-Buch, Berlin, Germany; ^2^Institute of Laboratory Medicine, Helios Klinikum Berlin-Buch, Academic Teaching Hospital, Berlin, Germany; ^3^Botanic Garden and Botanical Museum, Freie Universität, Berlin, Germany

## Abstract

**Background:**

The clinical features of a presumed capsaicin intoxication have not been reported so far. *Case Presentation*. A 27-year-old man took part in a qualifying for a competition in spicy food tolerance. During this qualifying, he swallowed 4 chili peppers type Bhut jolokia (about 1 million Scoville units) and other extremely spicy foods; the total amount of capsaicin ingested (roughly calculated retrospectively) accounted for at least 600 mg. After 2½ hours, the patient developed severe abdominal pain, which led to hospital admission. In contrast to the severe symptoms, clinical, laboratory, and imaging examinations (ultrasound and plain X-ray of the abdomen) did not reveal any significant abnormalities. Treatment with analgesics resulted in complete regression of the abdominal pain within 30 hours.

**Conclusions:**

The clinical picture in the view of pharmacological investigations on intestinal capsaicin infusions suggests that excessive doses of capsaicin can induce severe abdominal pain; the prolonged symptoms were probably due to the failure to vomit. Thus, a capsaicin intoxication must be considered in the differential diagnosis of an acute abdomen.

## 1. Background

Capsaicin is the pungent alkaloid present in chili peppers belonging to the species Capsicum. The content of capsaicinoids (90% of which constitutes of capsaicin and the almost equally potent derivative dihydrocapsaicin) varies not only among different genetic species of chili peppers but also depends on cultivating conditions [1, 2]. Pungency is mediated by interaction of capsaicinoids with the TRPV1 receptor (transient receptor potential vanilloid receptor subtype 1) [3], which is also activated by heat. Therefore, chili fruits induce the subjective sensation of burning. The intensity of spiciness is measured by Scoville heat units (SHUs), which indicate the number of times the substance has to be diluted in order that pungency is not perceived anymore [4]. Pure capsaicin measures 16 × 10^6^ SHUs, and in chili peppers, SHUs directly correlate with the capsaicin content of the fruits [1]. The hottest species is the genus “Trinidad Moruga Scorpion” [5] with up to 2 million SHUs, whereas the species “Bhut jolokia” is only slightly less spicy with 1–1.2 million SHUs [5, 6]. For comparison, Tabasco red sauce measures 2,500–5,000 SHUs [7].

There are numerous contests around the world in which participants (mainly young males) are challenged with extra-spicy food. However, the clinical significance of extensive consumption of extremely spicy foods leading to presumed capsaicin intoxication has—to our knowledge—not been reported in the medical literature so far.

## 2. Case Presentation

A 27-year-old male participated in a “qualifying” for a spicy food contest (“First Unofficial World Championship of Spicy Food Challenge”). He had been completely healthy previously except appendectomy some years earlier and did not take any medications. In particular, he had never experienced abdominal pain so far.

During the “qualifying,” the subject consumed the following food within 90 min (4.30–6 p.m.): 4 chilies of ghost peppers (Bhut jolokia), another chili (probably Habanero) with about 1 g of an extra spicy sauce of 294,400 SHUs, and 3 pieces of sausage each loaded with 6 g of spicy sauces at each level of 52,300, 99,800, and 294,400 SHUs, respectively, along with 3 slices of bread. In order to alleviate the burning in the mouth, the subject ingested chocolate drinks 3–5 times (quantity unknown).

The subject developed severe abdominal pain 2.5–3 hours after ingestion of the spicy food, partially crampy, otherwise permanent in nature associated with the sensation of warmth. He reported a feeling as if the abdomen would be extended and close to a rupture of the abdominal wall. Nausea and vomiting were completely absent, and vomiting had not occurred before admission. Due to the severe symptoms, he called on the emergency department of the hospital at 11 : 30 p.m. on the day of the qualifying. In contrast to the intensity of pain, the physical examination of the abdomen showed only slight tenderness of the upper abdomen, no muscular defense, and bowel sounds were normal. The body temperature was 36.7°C. Laboratory values were almost exclusively normal with exception of a slight elevation of lipase (174 U/l; normal value: <60 U/l) and lactate (3.75 mmol/l; normal value: <2.2 mmol/l). Blood cell count, C-reactive protein, electrolytes, transaminases, and creatinine were in the normal range. Soon after admission, an ultrasound investigation of the abdomen was performed, which did not show abnormalities.

**Figure 1:**
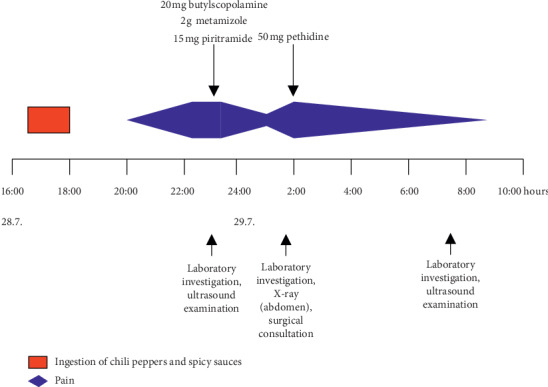
Time course of abdominal pain, diagnostic procedures, and medical therapy in the case presented.

Several analgesics were administered (Figure 1), which led to a temporary decrease in pain intensity (not quantitatively recorded, e.g., on a visual analogue scale), but subsequently, the symptoms markedly reoccurred associated with one single episode of vomiting about 12 hours after the ingestion. A plain radiograph of the abdomen did not show any pathological findings and, in a surgical consultation, there was no clinical evidence indicating necessary surgical interventions. The patient received additional opioid analgesics, finally resulting in an improvement. Two hours after admission, repeated laboratory measurements showed normalization of the blood lactate level (2.2 mmol/l) and a decrease of the lipase to 106 U/l, whereas all other parameters remained in the normal range.

At 8 a.m. the following day, the pain had almost completely disappeared and the clinical examination of the abdomen again did not reveal any pathological features. Laboratory values were completely in the normal range including lipase (30 U/l). Repeat ultrasound of the abdomen remained uneventful. Pain did not reappear within the following 24 hours, and the patient was discharged 35 hours after admission. Abdominal symptoms did not reappear over several months of follow-up.

Since (also in retrospect) there was no other overt cause for the abdominal pain except the ingestion of the extremely spicy food, the clinical picture was interpreted as a side effect of the consumption of excessive amounts of capsaicin.

## 3. Calculation of the Ingested Amount of Capsaicin

Since the total amount of capsaicin ingested by the patient could not be determined exactly, a rough calculation of the capsaicin content of the food administered was performed based on the information obtained from the organizer and the patient (Table 1); for conversion, 16 SHUs is equivalent to a capsaicin content of 1 mg/kg [1, 7].

### 3.1. Assumed Capsaicin Content of Chili Peppers

The patient ingested 4 chili peppers of the genus Bhut jolokia, of which the capsaicin content amounts for 1.0–1.2. million SHUs [5, 6] equivalent to 62.5 mg/g dry weight. To determine the dry weight of the chilies, 5 fresh chilies type Bhut jolokia were obtained from the Botanical Garden and Botanical Museum, Freie Universität Berlin, which were subsequently dried. The single mean dry weight accounted for 0.54 g (range 0.35–0.83 g), which represents 17% (mean) of the fresh weight. Based upon an assumed total dry weight of 2.16 g of the 4 chilies, the total amount of capsaicin of the 4 Bhut jolokia chilies ingested was 134 mg. Since the genus of the additional chili ingested is not precisely known, it was omitted from the calculation.

### 3.2. Assumed Capsaicin Content of Dressing (Sauces)

The preparation of the sauces was analyzed in detail with the organizer of the contest. Sauces of different pungency (800,000 SHUs up to 7.7 million SHUs) were diluted in tomato ketchup to give the different degrees of spiciness outlined in Table 1. Based on the amount of 6 g of sauces applied to each of the 3 pieces of sausage consumed at 3 different levels of pungency, the capsaicin content of the sauces taken with sausage summed up to about 500 mg (Table 1). Since the additional chili ingested was also loaded with the spiciest sauce of 294,400 SHUs (estimate 1 g), another 18 mg of capsaicin had to be added.

Thus, the total amount of capsaicin ingested by the patient during the qualifying procedure was estimated to be at least 600 mg based on this calculation.

## 4. Discussion and Conclusion

Chili peppers contain capsaicinoids in different concentrations, 90% of which are either capsaicin or dihydrocapsaicin. Both are equally spicy in pure form containing about 15-16 Mio SHUs, whereas the remaining capsaicinoids are less spicy (approximately 9-10 Mio SHUs) and account for less than 10% [1]. Ingestion of spicy chilies leads to severe burning in the oral cavity, larynx, and also frequently retrosternally; intensity of burning directly correlates to the spiciness of the respective chili genus. Severe burning and warmth after consumption of very spicy foods are well known in the general population and are rarely a reason for seeking medical attention.

Participants in spicy food contests frequently develop abdominal pain as confirmed by the organizer of multiple spicy food contests. However, with upcoming abdominal symptoms, the participants are asked to induce vomiting predominantly by rapidly drinking large amounts (1 to 2 l) of fluids [8], which probably prevent emptying of large capsaicin amounts from the stomach into the intestine. In the case presented here, the patient did not vomit at all until more than 12 hours after ingestion of the spicy food—probably too late for reducing the capsaicin load to the gut. It is hypothesized that the absence of vomiting led to the passage of the entire amount of capsaicin in the food down the whole gut and was thus responsible for the prolonged severe abdominal pain.

It could be shown experimentally in studies on the chemonociception in the gut that capsaicin infusion into the upper small intestine evoked pressure, cramps, pain, and nausea [9]. It seemed that intraduodenal infusion induced more pronounced symptoms than capsaicin given intrajejunally [9]. Symptoms in response to intestinal capsaicin administration were comparable to those reported by the patient presented here. Schmidt et al. [10] showed that intrajejunal infusion of capsaicin at rates between 100 and 1,000 *μ*g/min dose-dependently induced abdominal pain: based upon extraction of their data, absolute doses of 5–10 mg capsaicin given intrajejunally led to termination of the infusion due to abdominal pain [10].

Compared with the data of Schmidt and collaborators [10], the presumed amount of capsaicin ingested by the patient presented here was several times higher though a precise calculation of the capsaicin content was impossible for several reasons: the size of the chili peppers ingested is unknown; for calculation, an average weight was obtained from the freshly obtained chilies. In addition, the pungency and the capsaicin content of chilies are variable depending upon growing conditions [1]. Thus, the calculated capsaicin content in Bhut jolokia is somewhat speculative. On the other hand, the declared pungency of sauces is largely confirmed by analytical control experiments [7]. Therefore, the gross magnitude of the amount of capsaicin ingested by the patient seems to be reliable. These calculations support the concept that the severe symptoms are likely to be interpreted as capsaicin intoxication.

From the clinical standpoint, the most remarkable phenomenon was the discrepancy between almost unbearable pain on one hand and in contrast largely normal results regarding clinical findings, laboratory investigations, and imaging studies. Whether capsaicin was responsible for the small and short-lived elevation of blood lactate concentrations (which had returned to normal 2 hours after admission) is doubtful since capsaicin induces vasodilatation via stimulation of nitric oxide production [11]. On the other hand, it has been shown that chilies enhance “exogenic” lactate production by *Lactobacillus acidophilus* due to the increased metabolic activity of the bacterium [12], which could have been responsible for the short-lived increase in serum lactate. The mechanism for the short-lived and less than 3-fold lipase increase is unclear, but its clinical relevance is very doubtful: under experimental conditions, capsaicin has no effect on exocrine pancreatic secretion [13] and even improves the outcome in caerulein/bile acid-induced pancreatitis [14]. Furthermore, ultrasound investigations performed in the patient reported here did not reveal any abnormality of the pancreas.

Though it cannot be proven with certainty that the symptoms in the patient presented here were definitely caused by an overdose of capsaicin present in the different food stuffs ingested, this proposed mechanism is supported by a large number of evidence outlined above and—also important—in the absence of any other plausible cause for the severe abdominal pain. It seems obvious that excessive amounts of capsaicin can induce severe abdominal pain, thus mimicking the clinical picture of an acute abdomen. It remains to be established which threshold dose is required to generate such a symptomatology though the individual sensitivity is probably very variable [15] and may be additionally modulated by adaptation in subjects consuming constantly very spicy nutrients [16]. Since excessive capsaicin consumption has to be considered in the differential diagnosis of an acute abdomen, a careful dietary history should be obtained in such patients—particularly in the most probably affected group of young male patients, in which other organic causes of an acute abdomen are unlikely to be present.

## Figures and Tables

**Table 1:** Estimate of the amount of capsaicin presumably ingested by the patient (for further details see text).

Item	Pungency (SHU)	Presumed amount ingested (minimum) (g)	Corresponding capsaicin content (mg)
Chili peppers			
(i) 4 bhut jolokia	1.0 (−1.2) mio	2.16	134
(ii) 1 of unknown species	?	?	—

Sauces (with pieces of sausage)	52,300	18	59
	99,800	18	112
	294,400	18	330
(i) With chili pepper of unknown species	294,400	1	18

Total			653

## Data Availability

The data used to support the study are included within the article.

## Abbreviations

SHU: Scoville heat unit.

## References

[1] Collins M. D., Wasmund L. M., Bosland P. W. (1995). Improved method for quantifying capsaicinoids in capsicum using high-performance liquid chromatography. *HortScience*.

[2] Gonzales-Zamora A., Sierra-Campos E., Luna-Ortega J. G., Pérez-Morales R., Rodríguez Ortiz J. C., García-Hernández J. L. (2013). Characterization of different capsicum varieties by evaluation of their capsaicinoids content by high liquid chromatography, determination of pungency and effect of high temperature. *Molecules*.

[3] Caterina M. J., Schumacher M. A., Tominaga M., Rosen T. A., Levine J. D., Julius D. (1997). The capsaicin receptor: a heat-activated ion channel in the pain pathway. *Nature*.

[4] Scoville W. L. (1912). Note on capsicums. *The Journal of the American Pharmaceutical Association (1912)*.

[5] Bosland P. W., Coon D., Reeves G. (2012). ’Trinidad Moruga scorpion’ pepper is the world’s hottest measured Chile pepper at more than two million Scoville heat units. *HortTechnology*.

[6] Bosland P. W., Baral J. B. (2007). Bhut jolokia”-the world’s hottest known Chile pepper is a putative naturally occurring interspecific hybrid. *HortScience*.

[7] Forster S., Altmaier S. (2013). Simple and fast quantification of capsaicinoids in hot sauces using monolithic silica capillaries and LC-MS. *LCGC Europe*.

[8] Arens A., Ben-Youssef L., Hayashi S., Smollin C. (2016). Esophageal rupture after ghost pepper ingestion. *The Journal of Emergency Medicine*.

[9] Hammer J., Vogelsang H. (2007). Characterization of sensations induced by capsaicin in the upper gastrointestinal tract. *Neurogastroenterology & Motility*.

[10] Schmidt B., Hammer J., Holzer P., Hammer H. F. (2004). Chemical nociception in the jejunum induced by capsaicin. *Gut*.

[11] McCarthy M., Dinicolantonio J. J., O’Keefe J. H. (2015). Capsaicin may have important potential for promoting vascular and metabolic health. *Open Heart*.

[12] Sharma S., Jain S., Nair G. N., Ramachandran S. (2013). *Capsicum annuum* enhances L-lactate production by *Lactobacillus acidophilus*: implication in curd formation. *Journal of Dairy Science*.

[13] Schmidt P. T., Tornoe K., Poulsen S. S., Rasmussen T. N., Holst J. J. (2000). Tachykinins in the porcine pancreas: potent exocrine and endocrine effects via NK-1 receptors. *Pancreas*.

[14] Schneider L., Hackert T., Heck M. (2009). Capsaicin reduces tissue damage in experimental acute pancreatitis. *Pancreas*.

[15] Hammer J., Führer M. (2007). Intestinal chemo- and mechano-sensitivity: selective modification of small intestinal sensitivity by lipids. *Alimentary Pharmacology Therapeutics*.

[16] Führer M., Hammer J. (2009). Effect of repeated, long term capsaicin ingestion on intestinal chemo- and mechanosensation in healthy volunteers. *Neurogastroenterology and Motility: The Official Journal of the European Gastrointestinal Motility Society*.

